# From genomics to clinical practice: opportunities and challenges in addressing physical comorbidity in SMI

**DOI:** 10.3389/fpsyt.2025.1627629

**Published:** 2025-08-04

**Authors:** Junfeng Zhang, Xiaoling Liu, Bao Zhang, Xiaoyan Chen

**Affiliations:** ^1^ Department of Rehabilitation, Taihe Hospital, Hubei University of Medicine, Shiyan, Hubei, China; ^2^ Pediatric Department of Taihe Hospital, Hubei University of Medicine, Shiyan, Hubei, China; ^3^ School of Basic Medical Sciences, Hubei University of Chinese Medicine, Wuhan, China; ^4^ Key Laboratory of Traditional Chinese Medicine Resources and Traditional Chinese Medicine Formulas, Hubei University of Chinese Medicine, Wuhan, China

**Keywords:** psychiatry, polygenic risk scores (PRS), severe mental illness (SMI), mental health, genetics

## Introduction

We have closely examined the recent research by Kapell et al. published in The Lancet Psychiatry, which explores the significance of polygenic risk scores (PRS) for common medical diseases and comorbid physical health conditions in individuals with severe mental illness (SMI) (1). This groundbreaking research provides significant insights into the convergence of genetics, mental health, and physical comorbidities. The authors utilize polygenic risk scores to investigate the potential for identifying genetic predispositions to cardiovascular, metabolic, and immunological problems in patients with serious mental illness, highlighting the significant hereditary influence on physical health comorbidity. This opinion article will analyze the consequences of their findings, assess the merits and shortcomings of the applied approach, and explore prospective directions for future research in this quickly growing field.

## Significance of polygenic risk scores in SMI

The research by Kapell et al. is persuasive, providing strong evidence for the genetic foundations of concomitant physical problems in persons with severe mental illnesses, including schizophrenia, bipolar disorder, and major depressive disorder. Their results indicate that genetic vulnerability to cardiometabolic and autoimmune illnesses constitutes a substantial fraction of the physical health difficulties encountered by people with SMI. The observed impact sizes were similar to those found in the general population, regardless of conventional clinical and lifestyle risk factors.

This signifies a significant transformation in our approach to the convergence of mental and physical health. Integrating polygenic risk data may enable doctors to detect individuals at elevated risk for physical comorbidities earlier in the disease trajectory, thereby enhancing the prognosis for patients with SMI (2). Additionally, PRS may function as an innovative instrument for personalizing treatment and risk stratification, analogous to its current application in general populations for predicting the probability of prevalent diseases such as cardiovascular disease, diabetes, and autoimmune disorders (3).

Nonetheless, despite the persuasive nature of these findings, Kapell et al. recognize the limits of existing PRS techniques, especially the difficulties in using these genetic tools across varied populations. The practical utility of PRS is still ambiguous, particularly in non-European populations and regarding less common comorbidities. These problems underscore the necessity for ongoing research to enhance comprehension of the constraints and possibilities of polygenic risk scores in practical clinical environments. (**Box 1**).

Box 1 **What is a PRS?**

PRS is a quantitative measure that estimates an individual’s genetic predisposition to a particular trait or disease based on the cumulative effect of multiple genetic variants across the genome. Unlike monogenic disorders, which are caused by rare mutations in a single gene, most common complex diseases—such as cardiovascular disease, diabetes, and many psychiatric disorders—result from the combined influence of numerous genetic variants, each contributing a small effect.

The primary objective of PRS is to summarize the inherited risk conferred by a large number of common genetic variants (single nucleotide polymorphisms, SNPs) into a single score, which can then be used to stratify individuals according to their genetic risk for a disease or trait. In clinical research and, increasingly, in clinical practice, PRSs aim to enable earlier identification of individuals at high risk, inform preventative strategies, and guide personalized management.

The selection of genetic variants and the estimation of their effect sizes for PRS construction are typically based on data from large-scale Genome-Wide Association Studies (GWAS). GWAS investigate associations between millions of SNPs and traits of interest in large populations, allowing the identification of genetic variants that are statistically associated with disease risk. The effect sizes derived from GWAS summary statistics are then used as weights in calculating the PRS for each individual in an independent cohort. A PRS integrates information from thousands of genetic loci to provide a personalized estimate of disease risk, representing a powerful tool for precision medicine and population health research.

## Strengths and limitations

The study by Kapell et al. is distinguished by its extensive cross-cohort design, enhancing the generalizability of the results. By aggregating data from many cohorts, the scientists enhanced the statistical power of their studies and addressed variability in patient populations. This methodology is a notable advantage, as it strengthens the validity of their conclusions and offers a more thorough comprehension of the influence of genetics on comorbid physical health issues in SMI.

A principal strength of the study is its focus on employing PRS to forecast not only the existence of physical ailments but also the severity of these illnesses in individuals with SMI. This presents the opportunity to enhance clinical decision-making, advancing from basic risk categorization to more sophisticated risk profiling. This facilitates personalized interventions that may alleviate the physical health burden experienced by those with severe mental illness.

Nevertheless, various restrictions warrant additional examination. The study illustrates the hereditary influence on physical health comorbidity in serious mental illness, although the variance accounted for by polygenic risk scores is limited. This prompts significant enquiries regarding the practical efficacy of genetic profiling when utilized independently. Notwithstanding the encouraging findings, PRS constitutes but a small portion of the observed variance in physical health outcomes, indicating that environmental, behavioral, and treatment-related factors may significantly influence patient outcomes (4). Consequently, PRS ought to be seen as a component of a broader framework encompassing lifestyle factors, medical history, and treatment interventions.

Furthermore, as noted by Kapell et al., the existing PRS models predominantly rely on data from populations of European ancestry. This imposes considerable constraints on the generalization of the findings to various ancestral groups. In light of the increasing acknowledgement of genetic variation, it is essential that forthcoming research includes more diverse global cohorts to guarantee that polygenic risk scores are universally relevant and equitable among various populations.

## Future directions for research

Although Kapell et al.’s research offers significant insights into the genetic underpinnings of comorbid physical health issues in severe mental illness, the area remains nascent. Several critical domains must be prioritized for future investigation to properly harness the promise of PRS in clinical practice.

Initially, longitudinal studies are required to monitor individuals over an extended period. Longitudinal designs will enable researchers to investigate the temporal dynamics of genetic risk, assessing whether polygenic risk scores predict future physical health outcomes in individuals with SMI. This will elucidate the interaction between genetic risk and environmental factors in influencing disease progression and the development of comorbidities.

Secondly, research ought to aim at integrating genetic risk with environmental and treatment-related variables. Although genetic susceptibility is undeniably significant, it is equally crucial to acknowledge the impact of modifiable factors, like food, physical exercise, and medication adherence, on the emergence of physical comorbidities. The integration of genetic and environmental risk evaluations may result in more accurate and individualized treatment approaches that consider both hereditary and lifestyle factors contributing to physical health disparities in SMI.

Third, research should focus on enhancing the genetic variety of the populations examined. Current PRS models are primarily derived from European populations, perhaps restricting their relevance to other ethnic and racial groupings. Subsequent study must incorporate more heterogeneous cohorts to guarantee that genetic risk scores are pertinent and efficacious across various populations. This will also address health equality issues, as individuals from varied origins may possess differing genetic susceptibilities to physical ailments.

Ultimately, interdisciplinary collaboration is essential for the progression of this domain. Geneticists, psychiatrists, physicians, and epidemiologists must collaborate to create and enhance instruments for forecasting physical health outcomes in individuals with severe mental illness. Collaboration is essential to fully realize the potential of genetic risk assessment, thereby assisting persons with severe mental illness globally.

## Addressing ancestry limitations in polygenic risk scores

A well-recognized limitation of current PRS models is their restricted applicability across diverse ancestral populations, as most GWAS and subsequent PRS development have predominantly involved individuals of European descent. This bias can substantially reduce the predictive performance and clinical utility of PRS in non-European groups, potentially exacerbating health disparities (3, 5).

To overcome this challenge, several strategies are being actively pursued. Firstly, increasing the ancestral diversity of GWAS cohorts is essential to ensure that genetic risk prediction is accurate and equitable across populations. Large-scale international initiatives such as the H3Africa Consortium (6) and the All of Us Research Program (7) are making significant progress in recruiting participants from underrepresented populations and generating high-quality genomic data. These efforts are expected to improve the representation of diverse ancestries and enhance the transferability of PRS. Secondly, methodological advances are underway to develop trans-ancestry or ancestry-agnostic PRS models. These approaches involve the integration of genetic data from multiple populations and the application of statistical methods that account for differences in linkage disequilibrium and allele frequencies across ancestries. Early evidence suggests that such methods can improve the accuracy and generalizability of PRS in diverse groups (8, 9).

In summary, while ancestry-related limitations remain a significant concern, ongoing research and collaborative initiatives are providing practical solutions. Continued efforts to increase GWAS diversity and develop more sophisticated PRS methodologies will be crucial for ensuring that the benefits of genomic medicine are accessible to all, regardless of genetic background.

## Conclusion

In conclusion, the research conducted by Kapell et al. signifies a substantial advancement in comprehending the genetic foundations of comorbid physical health issues associated with severe mental illness. Although their findings offer significant potential for enhancing risk assessment and clinical decision-making, concerns persist regarding generalizability, the limited variance accounted for by polygenic risk scores, and the necessity for deeper integration of genetic, environmental, and treatment-related components. Future research must emphasize longitudinal studies, the incorporation of varied cohorts, and the creation of personalized, integrated risk models that consider both genetic and environmental factors (5). Ongoing research and collaboration may facilitate the incorporation of polygenic risk scores into clinical practice, serving as a potent instrument for addressing the intricate health requirements of patients with severe mental illness and enhancing their physical and mental well-being.
